# Observation of the therapeutic effect of apatinib in advanced platinum-resistant recurrent epithelial ovarian cancer

**DOI:** 10.1186/s13048-022-01055-4

**Published:** 2023-02-23

**Authors:** Zhongmian Pan, Zhongbin Luo, Hongying He, Yujie Chen, Bingbing Zhao, Zhijun Yang, Li Li

**Affiliations:** 1 Department of Gynecology and Oncology, Guangxi Medical University Cancer Hospital, 71 Hedi Road, Nanning, 530021 China; 2 Department of Obstetrics and Gynecology, Liuzhou Workers Hospital, Liuzhou, China; 3 Department of Obstetrics and Gynecology, Liuzhou People’s Hospital, Liuzhou, China

**Keywords:** Apatinib, Advanced recurrent epithelial ovarian cancer, Angiogenesis inhibitor, CA125

## Abstract

**Background:**

Apatinib is an oral anti-angiogenic drug that mainly targets vascular endothelial growth factor receptor 2 (VEGFR-2) and is widely used in a variety of solid tumours. The purpose of this study is to evaluate the clinical efficacy and safety of apatinib in patients with advanced platinum-resistant relapsed epithelial ovarian cancer (EOC).

**Methods:**

A retrospective analysis was performed, the clinical data of patients with stage IIIC-IV platinum-resistant relapsed EOC between January 2014 and May 2018 were collected. The objective response rate (ORR), disease control rate (DCR), progression-free survival (PFS), and overall survival (OS) were reviewed and evaluated. The propensity score matching (PSM) method was used to determine the final case data included in this study.

**Results:**

According to 1:2 propensity matching, 108 patients were finally taken into account: 36 in the apatinib group and 72 in the control group. The follow-up ended in January 2019, and the median follow-up time was 28 months. In the apatinib group, ORR was 30.56% and DCR was 66.67%, whereas in the control group, ORR was 16.67% and DCR was 44.44%. In the apatinib group, median PFS was 6.0 months (95% CI 3.69–8.31) and median OS was 15.8 months (95% CI 6.99–24.6), while in the control group, median PFS was 3.3 months (95% CI 2.44–4.16) and median OS was 9.2 months (95% CI 6.3–12.06); the difference was statistically significant (*P* < 0.05). Apatinib was more effective than conventional chemotherapy in reducing the risk of PFS [HR 0.40 (95% CI 0.22–0.76), *P* = 0.0017] and OS [HR 0.40 (95% CI 0.21–0.73), *P* = 0.002]. Multivariate Cox analysis showed that the course of treatment and decrease in serum CA125 levels are independent risk factors for PFS in patients, while apatinib, the length of treatment course and the location of the lesion are independent risk factors for recurrence affecting the OS of patients. The main grade 3–4 adverse events in the apatinib group were hypertension, hand-foot syndrome, and oral mucosal ulcers, and all adverse events were controllable.

**Conclusion:**

Apatinib was found to be both safe and effective in patients with advanced platinum-resistant relapsed EOC. More in-depth clinical research and applications should be carried out.

## Introduction

Malignant ovarian tumours are one of the most common gynaecological tumours. According to statistics from the Global Cancer Center in 2018, there were 295,414 new cases of malignant ovarian tumours and 184,199 deaths, accounting for more than half of the new cases [1]. Approximately 85–90% of primary ovarian cancer patients with ovarian malignancies are diagnosed with epithelial ovarian cancer (EOC), including serous, mucinous, endometrioid and clear cell EOC [2, 3]. According to the statistics of ovarian cancer data in the United States in 2018, nearly 80% of patients were diagnosed with advanced stage, that is, stage III-IV EOC [4]. The current main treatment methods are surgery and platinum-based adjuvant chemotherapy, but the overall curative effect is poor. Approximately 70% of patients will experience disease recurrence. The types of recurrence are mainly platinum-sensitive, platinum-resistant or platinum-refractory recurrence. Recurrence typically occurs more than 6 months after the completion of the last platinum-based treatment. Progression is considered platinum-sensitive recurrence, and progression during platinum-containing treatment or within 6 months after the completion of platinum-based treatment is defined as platinum-refractory or platinum-resistant recurrence. Most recurrent ovarian cancers eventually develop platinum-resistant recurrence, leading to a poor response to traditional cytotoxic chemotherapy [5, 6], which is the cause of death in patients with ovarian cancer and a serious threat to women’s health. Clinical work urgently needs to explore more effective treatment options.

The continuous formation of abnormal blood vessels is considered to be a key step in tumour growth and metastasis [7]. Thus, it is an attractive treatment strategy for ovarian cancer. Vascular endothelial growth factor (VEGF) is one of the most effective angiogenic factors and binds to three vascular endothelial growth factor receptors (VEGFRs), VEGFR-1, VEGFR-2 and VEGFR, in a variety of solid tumours with important carcinogenic effects. Studies have shown that compared with normal ovarian tissue, the expression level of VEGF in EOC tissue is significantly higher, which is closely related to the formation of ascites, the degree of histological malignancy, distant tumour metastasis, and reduced survival rates. In addition, compared with platinum-sensitive tumours, platinum-resistant ovarian cancer has significantly higher levels of the growth factor receptors PDGFR-beta and VEGFR2, which are related to angiogenesis. VEGFR-2 is an important target in the endothelium. Its expression on cells is the main mediator of VEGF-induced angiogenesis [8–10]. Therefore, blocking VEGFR-2 to inhibit the VEGF signal transduction pathway has become a promising strategy to inhibit abnormal angiogenesis and treat platinum-resistant relapsed EOC. In recent years, many studies on angiogenesis inhibitors (AIs) such as bevacizumab have confirmed the application of this strategy in advanced recurrent ovarian cancer, which has achieved gratifying results. AIs have been approved for use in the first-line or second-line treatment of advanced or recurrent ovarian cancer. Bevacizumab for maintenance therapy [11, 12] is the first targeted drug currently approved for platinum-resistant recurrent ovarian cancer [13]. However, it is expensive, and most patients with advanced platinum-resistant recurrent ovarian cancer cannot afford this financial pressure.

Apatinib mesylate (Apa) is a kind of oral AI that was independently developed by China that highly selectively inhibits VEGFR-2 and slightly inhibits c-Kit and c-Src tyrosine kinases [14]. Malignant tumours show encouraging antitumour activity and tolerable toxicity. By competitively binding to the kinase domain of VEGFR-2, it inhibits the VEGF signalling pathway and thus inhibits angiogenesis. In animal experiments and clinical trials, there was direct evidence showing that apatinib can normalize tumour blood vessels, correct tumour blood concerns and the hypoxic tumour microenvironment and is beneficial to the improvement of other related chemotherapeutic drugs in tumour delivery. Apatinib, as an AI administered orally [15], has the advantages of high bioavailability and convenient medication and can significantly improve patient compliance with medication [16]. The purpose of this article is to use propensity score matching (PSM) to eliminate confounding factors and to evaluate the efficacy and safety of the targeted drug apatinib compared with conventional chemotherapy in the treatment of advanced recurrent EOC, in view of the clinical work in China to provide different treatment strategies for platinum-resistant recurrent ovarian cancer.

## Materials and methods

### General information

To retrospectively analyse the clinical data of patients with advanced EOC with tumour platinum-drug resistance and recurrence who were admitted to the Affiliated Tumour Hospital of Guangxi Medical University, Liuzhou Workers’ Hospital and Liuzhou People’s Hospital from January 2014 to June 2018. The inclusion criteria were as follows: (1) patients with EOC diagnosed by pathology; (2) patients with at least one lesion for imaging measurement such as computed tomography (CT), or magnetic resonance imaging (MRI) p; (3) patients with advanced tumour recurrence, stage IIIc-IV according to International Federation of Gynecology and Obstetrics (FIGO) surgery-pathological staging; (4) patients who underwent ovarian tumour cytoreduction surgery or standard first-line platinum-based chemotherapy and now have uncontrolled or relapsed platinum-resistant disease after second-line or n-line chemotherapy; and (5) patients treated with oral apatinib who met the ethical requirements and signed the relevant informed consent form. The exclusion criteria were as follows: (1) patients with severe cardiovascular and cerebrovascular events in the past 6 months; (2) patients with irreversible liver and kidney damage; and (3) patients with hypertension who cannot be controlled by drugs. According to the inclusion and exclusion criteria, 144 patients were finally included in the study group. According to different treatment plans, the patients were divided into the apatinib group (44 patients) and the control group (100 patients). The apatinib group was treated with apatinib combined with chemotherapy, and the control group was treated with second-line and above conventional chemotherapy drugs after recurrence.

### Treatment methods

In the apatinib group, the oral targeted drug apatinib mesylate (Jiangsu Hengrui Pharmaceuticals Co., Ltd.) was administered as follows: initial dose 500 mg/time, qd, oral administration; when side effects such as high blood pressure or serious peeling of the hands and feet occur, the dose was adjusted to 250 mg/time qd orally; apatinib was combined with chemotherapy, and the chemotherapy regimen was determined by the medical treatment (etoposide in 2 cases, accounts for 4.5%. paclitaxel in 10 cases with weekly, accounts for 22%. gemcitabine combined with oxaliplatin in 8 cases, accounts for 18%, 15 cases in PLD combined with carboplatin,accounts for 34%, and 9 cases in irinotecan combined with pemetrexed,accounts for 20%). Patients in the control group were given conventional second-line therapy after recurrence, and the regimen was determined by the physician (etoposide 10 cases, accounts for 10%, paclitaxel weekly therapy 22 cases, accounts for 22%, gemcitabine combined with oxaliplatin 20 cases, accounts for 20%, PLD combined with carboplatin 27 cases accounts for 27%,and irinotecan combined with pemetrexed 21 cases,accounts for 21%).

### Observation indicators

secondary research indicators were overall survival (OS) and objective response rate (ORR). 3) After drug treatment, when the tumour marker CA125 decreased between the two groups of patients (the normal value of CA125 was < 35 U/m), and shrinkage with measurable tumor lesions. The Gynecological Cancer Society (GCIG) believes that a decrease in the level of CA125 by at least 50% or a reduction in lesions of 30% or more indicates a good treatment effect [17]. 4) The safety indicator was the incidence of adverse events.

### Efficacy evaluation criteria

The evaluation of the efficacy of oncology drugs was based on the response evaluation criteria of solid tumours (RECIST 1.1) [18]. (1) Complete remission (CR): It is required that all recurrences disappear and the short axis value of any pathological lymph nodes is less than 10 mm. (2) Partial remission (PR): The sum of the radius of all target lesions is reduced by at least 30% compared with the sum of the critical radius as a reference. (3) Stable disease (SD): Those who have not met the standard for PR but have not reached the standard for progressive disease (PD). (4) PD: The total radius of all target lesions is increased by at least 20% or new lesions appear. ORR = (CR + PR) number of cases/total number of cases× 100%; disease control rate (DCR) = (CR + PR + SD) number of cases/total number of cases× 100%. PFS was defined as the time from the start of medication to the recurrence of disease, disease progression or other causes of death. The basis for evaluating adverse reactions refers to the Common Terminology Criteria for Adverse Events (CTCAE 4.0).

### Follow-up

The follow-up method was performed in the outpatient clinic and by telephone, and the follow-up date ended in January 2019. The main outcome measure, PFS, was defined as the time from the recurrence of the tumour or the progression of the disease after the use of second- and third-line drugs and the beginning of apatinib or chemotherapy to the recurrence of earlier disease progression or death. OS was defined as the time from the start of retreatment to the death of the patient or the last follow-up.

### Statistical methods

The statistical software SPSS 22.0, GraphPad Prism 8.0.2, and R 2.15.3 and the corresponding R plug-in and PS Matching 3.04 software were used to process all the data in this study. The PSM analysis method refers to the study of West et al. [19], which uses the chi-square test for count data, sets the case ratio to 1:2, sets the calliper value to 0.1, uses the t-test or rank-sum test for statistics after matching measurement data according to whether they obey a normal distribution, and uses the chi-square test for count data. The survival data were analysed by the Kaplan-Meier method to draw survival curves, and the log-rank test was used to compare the survival curves. The hazard ratio (HR) and its 95% confidence interval (CI) were calculated. A Cox proportional hazards regression model was used for multivariate analysis, with *P* < 0.05 indicating that the difference was statistically significant.

## Results

### Comparison of the baseline data of the apatinib group and control group before and after PSM

**Fig. 1:**
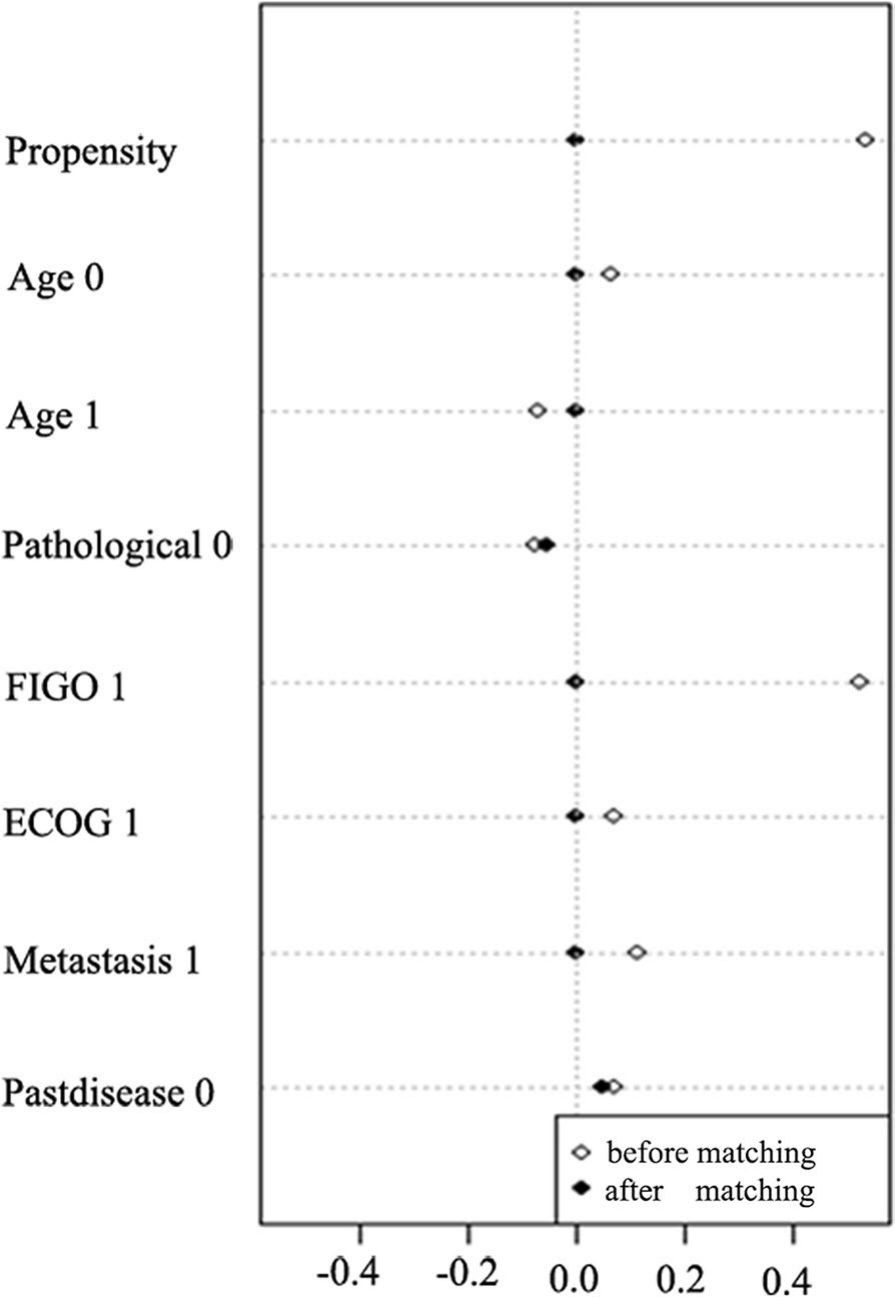
Standardization difference line chart and univariate scatterplot

According to the inclusion and exclusion criteria, the retrospective analysis of clinical data included a total of 144 patients, including 44 patients in the apatinib group and 100 patients in the control group. The analysed baseline characteristics of the patients in the two groups included age, pathological type, tumour FIGO staging, Eastern Cooperative Oncology Group (ECOG) score, location of the recurrent lesions, and whether they had underlying diseases. The first two groups of PSM patients had significant differences in tumour staging and the location of the recurrent lesions when the disease recurred (*P* < 0.05), indicating that the two groups were imbalanced in terms of these two factors. Propensity matching was performed based on the above baseline characteristics according to a 1:2 case ratio. After PSM matching, a total of 108 patients were finally included in this study, 36 in the apatinib group and 72 in the control group. The standardized difference change line chart and univariate scatterplot show (Fig. 1) that the standard difference of each covariate was significantly reduced after matching, and the univariate SD scatter plot shows that the standard difference after matching is basically concentrated at approximately 0 (< 10%), indicating that the variables are balanced and the matching effect was good. There was no significant difference in the baseline data of the two groups of patients (*P* > 0.05) (see Table 1).

**Table 1:** Comparison of the baseline data of the two groups before and after PSM

	Before PSM			After PSM		
Variable	Apatinib group (***n*** = 44)	Control group (***n*** = 100)	P	Apatinib group (***n*** = 36)	Control group (***n*** = 72)	P
Age, years ($\overline{x}$ ± s)	52.48 ± 9.96	51.76 ± 9.42	0.69	52.28 ± 8.32	53.39 ± 8.32	0.515
Histology			0.786			0.991
Serous	32	77		28	54	
Endometrioid	4	8		3	6	
Mucinous	3	9		2	6	
Clear	1	2		1	2	
Other	4	4		2	4	
FIGO Stage			0.03			0.297
IIIc	24	79		23	53	
IV	20	21		13	19	
ECOG performance status			0.776			0.988
0	10	20		8	16	
1	17	45		16	33	
2	17	35		12	23	
Recurrent lesions			0.033			0.637
In the pelvic cavity	11	24		10	17	
Far beyond the pelvis	33	76		26	55	
Basic illness			0.592		0.779	
Yes	17	34		13	28	
No	27	66		23	44	

### Patients’ completed treatment status and short-term efficacy evaluation

The follow-up time was as of January 2019, and the median follow-up time was 28 months. Thirteen patients in the apatinib group adjusted the dose of apatinib due to adverse events (AEs) such as intolerable hypertension and severe hand-foot syndrome. A total of 250 mg was taken orally every day, and the conventional chemotherapy group was treated with different second-line or third-line schemes after the evaluation of tumour recurrence. A total of 233 cycles of chemotherapy were completed before tumour progression was evaluated again. The clinical efficacy of all patients could be evaluated after drug treatment. In the apatinib group, 1 patient had CR (2.78%), 10 had PR (27.78%), 13 had SD (36.11%), and 12 had PD (33.33%). In the control group, 1 patient had CR (1.38%), 11 patients had PR (15.28%), 20 patients had SD (27.78%), and 40 patients had PD (55.56%).

The path for the application of the tumor recurrence significant reduction or stabilize lesions have obvious effect (*P* < 0.05) (see Table. 2).

**Table 2:** Changes after treatment between two groups of patients with lesions

Group	Narrow OR stable lesions	Lesions increase	All cases
Apatinib group (n)	24	12	36
Control group (n)	32	40	72
P			0.029

There was no significant difference in serum CA125 levels between the two groups of patients before drug intervention (*P* > 0.05). After drug intervention, the serum CA125 levels of patients in the apatinib group were significantly lower than those in the control group (*P* < 0.05). After PSM analysis, apatinib significantly reduced the serum CA125 levels of patients (*P* < 0.05), while the serum CA125 levels of patients in the control group increased after treatment (*P* < 0.05). Comprehensive analysis shows that compared with conventional chemotherapy, apatinib can significantly reduce the CA125 levels of patients and reduce the tumour burden of recurrent lesions (see Table 3).

**Table 3:** Comparison of CA125 levels before and after treatment between the two groups (x ®± s)

Group	CA125 changes		
	Before treatment	After treatment	P
Apatinib group	929.03 ± 1694.71	408 ± 565.50	0.032
Control group	700.52 ± 1332.16	1091.084 ± 1928.68	0.006
P	0.446	0.005	

**Fig. 2:**
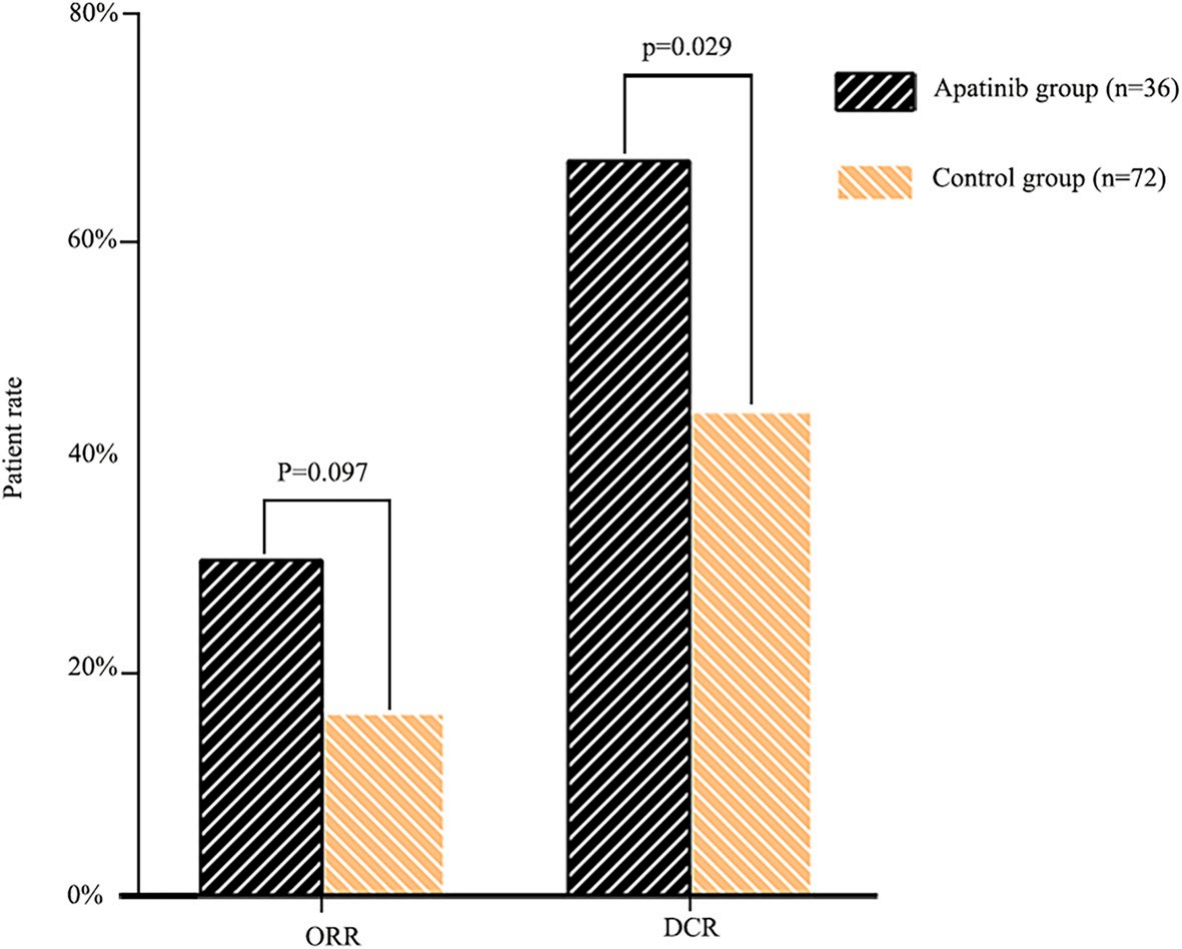
ORRs and DCRs of the two groups of patients

**Fig. 3:**
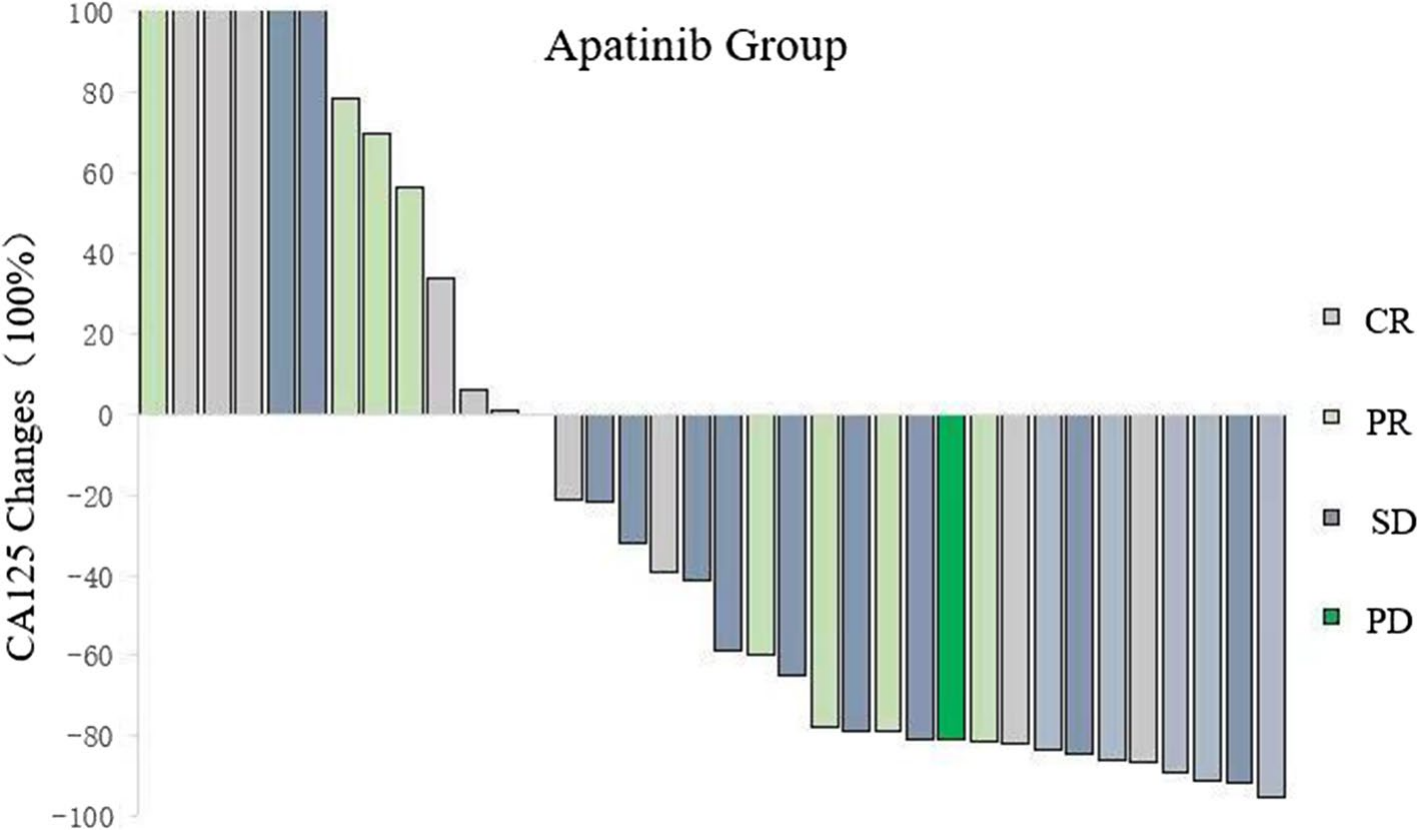
Changes in the after treatment (in CR, PR, SD, and PD)

The DCR of the apatinib group was significantly improved compared with that of the control group (66.67% vs. 44.44%), *P* < 0.05, while for the ORR, there was no significant difference between the two groups of patients (30.56% vs. 16.67%), *P* > 0.05 (see Fig. 2 and Fig. 3 Changes in the after treatment).

### Comparative analysis of PFS results between the two groups of patients

**Fig. 4:**
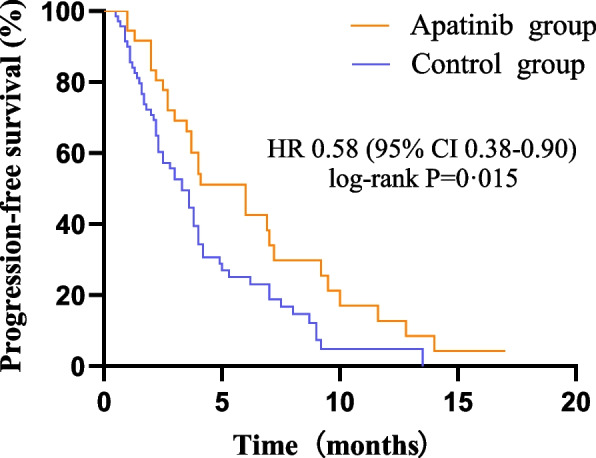
Comparison of PFS between the two groups

The PFS results were analysed, and the median PFS of the apatinib group was 6.0 months (95% CI 3.69–8.31), while the median PFS of the control group was 3.3 months (95% CI 2.44–4.16). The difference was statistically significant (*P* < 0.05), and the use of apatinib after disease recurrence in patients with advanced recurrent ovarian cancer can reduce the risk of disease progression compared with conventional chemotherapy [HR 0.58 (95% CI 0.38–0.90), *P* = 0.015] (see Fig. 4).

### Comparative analysis of the OS results between the two groups

**Fig. 5:**
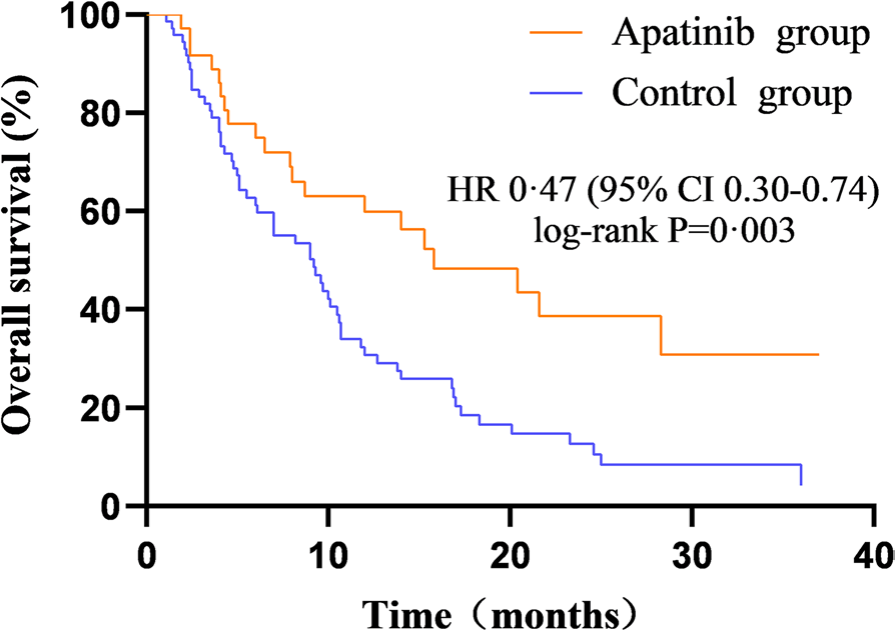
Comparison of PFS between the two groups

The OS results of the two groups of patients were also analysed. The median OS of the apatinib group was 15.8 months (95% CI 6.99–24.6), and the median OS of the conventional chemotherapy group was 9.2 months (95% CI 6.3–12.06). The difference was statistically significant (*P* < 0.05). The use of apatinib after disease recurrence in patients with advanced recurrent ovarian cancer can reduce the risk of death in patients compared with conventional chemotherapy [HR 0.47 (95% CI 0.30–0.74), *P* = 0.003] (see Fig. 5).

### Single-factor analysis of PFS and OS in the two groups of patients

**Fig. 6:**
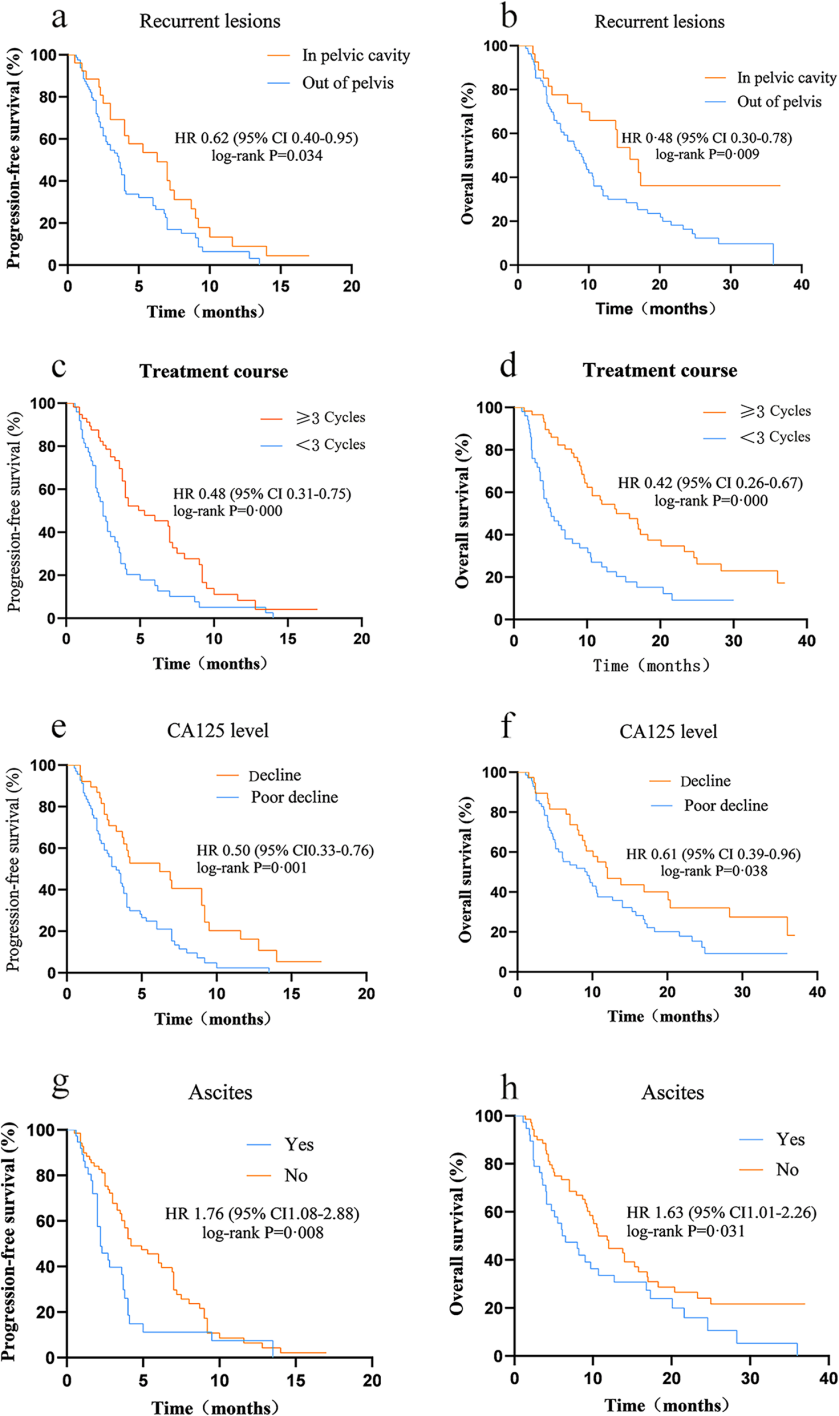
Single-factor analysis of factors related to PFS and OS

The above data analysis shows that apatinib can improve the survival prognosis of patients with recurrent ovarian cancer. In addition, we analysed other single factors that may affect PFS in platinum-resistant recurrent ovarian cancer, and the specific results are presented in Table 3. Among them, location of the lesion at the time of tumour recurrence, decrease in serum CA125 levels, course of medication, and presence or absence of ascites at the time of recurrence were all related single factors affecting the patient’s PFS and OS (*P* < 0.05) (see Fig. 6). However, age, tumour stage, pathological type, and presence of underlying diseases were not significantly related to PFS or OS (*P* > 0.05).

The use of apatinib after disease recurrence in patients with advanced recurrent ovarian cancer can reduce the risk of death in patients compared with conventional chemotherapy [HR 0.47 (95% CI 0.30–0.74), *P* = 0.003] (see Fig. 5).

### Single-factor analysis of PFS and OS in the two groups of patients

The above data analysis shows that apatinib can improve the survival prognosis of patients with recurrent ovarian cancer. In addition, we analysed other single factors that may affect PFS in platinum-resistant recurrent ovarian cancer, and the specific results are presented in Table 3. Among them, location of the lesion at the time of tumour recurrence, decrease in serum CA125 levels, course of medication, and presence or absence of ascites at the time of recurrence were all related single factors affecting the patient’s PFS and OS (*P* < 0.05) (see Fig. 6). However, age, tumour stage, pathological type, and presence of underlying diseases were not significantly related to PFS or OS (*P* > 0.05) (Table 4).

**Table 4:** Single-factor analysis of PFS and OS in the two groups of patients

	n	PFS (months) 95% CI	P	OS (months) 95% CI	P
Ages (years)			0.676		0.858
≥ 50	45	3.60(3.01–4.19)		8.70 (4.42–12.98)	
< 50	63	4.00(3.42–4.58)		10.00 (8.52–11.48)	
Recurrent lesions			0.034		0.009
In pelvic cavity	27	6.3(2.48–11.51)		15.8(12.53–19.07)	
Out of pelvis	81	3.60(2.95–4.25)		9.00(7.10–10.90)	
CA125 level			0.001		0.038
Decline	38	7.0(3.86–10.15)		12.00(7.63–16.37)	
Poor decline	70	3.0(1.91–4.09)		9.30(5.10–13.51)	
Adverse reactions			0.089		0.540
≥ 3	27	3.60(2.94–4.26)		9.30(6.55–12.05)	
None or ≤ 2	81	4.20(2.43–5.97)		10.60(7.95–13.25)	
FIGO Stage			0.912		0.667
IIIC	76	3.80(3.43–4.17)		12.70(4.47–20.93)	
IV	32	3.30(1.84–4.76)		9.70(7.87–11.53)	
Treatment course			0.000		0.000
≥ 3 cycles	58	6.2(4.02–8.38)		14.00(8.78–19.22)	
< 3 cycles	50	2.2(1.87–2.53)		5.10(2.87–7.33)	
Surgery again after recurrence			0.046		0.065
Yes	37	6.0(3.68–8.32)		14.0(9.28–18.72)	
No	71	3.6(2.69–4.51)		9.00(7.15–10.85)	
Histology			0.939		0.886
Serous	82	3.80(3.41–4.06)		10.50(8.74–12.26)	
Non-serous	26	3.80(1.93–5.67)		9.30(7.12–11.48)	
Ascites			0.008		0.031
Yes	38	2.2(1.41–2.98)		6.10(2.32–9.88)	
No	70	4.2(2.47–5.93)		10.7(8.40–13.00)	
Basic illness			0.163		0.729
Yes	41	3.60(2.74–4.46)		9.30(6.94–11.66)	
No	67	4.00(3.46–4.54)		10.70(7.75–13.65)	

### Analysis of the multifactor cox regression model for PFS and OS

#### Multivariate regression analysis of PFS in patients

After the above analysis, the important factors affecting PFS and prognosis were analysed by a multifactor Cox regression model. Taking the PFS time as the dependent variable and the significant indexes in the single-factor analysis as the independent variables, the input method was used for multifactor Cox regression analysis. The results show that the independent factors affecting PFS were the treatment course and changes in serum CA125 levels. That is, the shorter the course of treatment or the greater the decline in serum CA125 levels, the greater the risk of recurrence of tumour disease progression was (see Table 5).

**Table 5:** Analysis of the multivariate Cox regression model for late recurrent E

	B	SE	Wald	HR (95% CI)	P
Treatment course	0.561	0.232	5.837	1.752(1.112–2.762)	0.016
CA125 level	0.562	0.245	5.268	1.755(1.086–2.837)	0.022
Recurrent lesions	0.181	0.271	0.446	1.198(0.705–2.037)	0.504
Adverse reactions	0.362	0.288	1.579	1.436(0.817–2.526)	0.209
Surgery	0.055	0.260	0.045	1.056(0.635–1.758)	0.833
Ascites	−0.276	0.277	0.993	0.758(0.440–1.306)	0.319
Basic illness	0.140	0.237	0.352	1.151(0.724–1.830)	0.553

#### Multivariate regression analysis of OS in patients

With death and OS time as dependent variables and significant indicators in the univariate analysis as independent variables, multivariate Cox regression analysis using the input method showed that independent risk factors affecting OS include course of drug treatment after tumour recurrence (with chemotherapy course ≥3 courses as the reference category), location of the lesion at the time of recurrence (with the recurrence of the pelvic lesion as the reference category), and whether apatinib was used (with the control group as the reference category). That is, the use of apatinib after the relapse of platinum-resistant disease is an independent factor that significantly improves the prognosis of patients and reduces mortality. The longer the medication time, the longer the patient’s OS time is. The patient’s prognosis is also related to the type of tumour recurrence, and patients with distant metastases outside the pelvis are at higher risk of death (see Table 6).

**Table 6:** Multivariate Cox analysis of factors affecting late recurrent EOC

	B	SE	Wald	HR (95% CI)	P
Treatment course	1.018	0.252	16.279	2.768(1.688–4.539)	0.000
Apatinib	−0.968	0.285	11.552	0.380(0.217–0.644)	0.001
Recurrent lesions	0.626	0.305	4.206	1.870(1.028–3.403)	0.04
Surgery	0.156	0.273	0.326	1.168(0.685–1.994)	0.568
CA125 level	0.009	0.274	0.001	1.009(0.590–1.726)	0.974
Ascites	−0.170	0.248	0.326	0.844(0.518–1.373)	0.494

### Analysis of adverse reactions

**Fig. 7:**
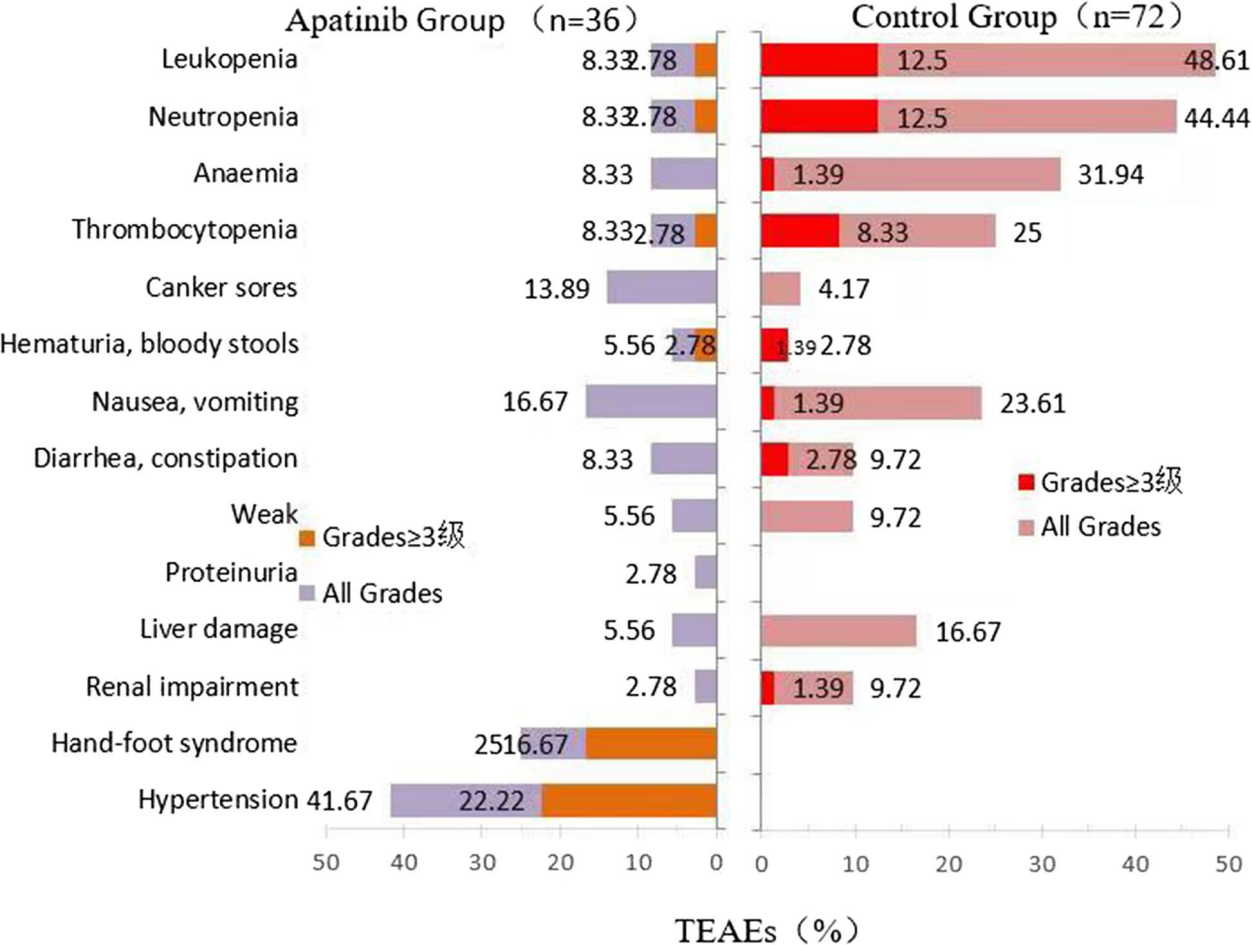
Comparison of adverse reactions between the two groups

The incidence of AEs including hypertension, hand-foot syndrome, etc. was significantly higher in the apatinib group than in the normal chemotherapy group, while the incidence of haematological toxicity was lower, and the differences were statistically significant (*P* < 0.05). AEs such as gastrointestinal reactions, nausea, vomiting and related important organ function impairments were compared between the apatinib and conventional chemotherapy groups, and the difference was not statistically significant (*P* > 0.05). The incidence of grade 3–4 blood pressure elevation events during the administration of apatinib was 21.28%. A review and analysis of clinical data showed that oral antihypertensive drugs or adjustment of the dosage of apatinib can control and stabilize blood pressure. There were no fatal events related to adverse reactions, and all AEs could be controlled (see Fig. 7).

## Discussion

In recent years, although important progress has been made in the treatment of EOC, because most patients are already at an advanced stage when they are diagnosed and because of the high risk of metastasis and recurrence, approximately 70% of patients with advanced ovarian cancer will develop disease recurrence and may eventually develop platinum-resistant recurrence. This is the main reason that EOC mortality ranks first in female malignant tumours [2, 4]. The choice of treatment methods for advanced recurrent EOC, improving the quality of life and improving patient survival prognosis are still important problems facing clinical work.

Abnormal angiogenesis is the anatomical and physiological basis of tumour growth and metastasis and promotes the abnormal proliferation and distant metastasis of tumours. The degree of formation directly affects the survival and prognosis of ovarian cancer patients. VEGF is one of the key factors that stimulates angiogenesis. It is released by cancer cells and binds to endothelial cell receptors (i.e., VEGFR) to stimulate tyrosine kinase activity, which in turn stimulates downstream signal transduction and endothelial cell activation and promotes the generation of abnormal blood vessels [20]. With the development of modern biology, anti-angiogenesis strategies have become an effective method to overcome recurrent ovarian cancer. AIs bring new hope to patients. A variety of AIs that have been successfully developed can act on different targets and disrupt abnormal angiogenesis-related pathways [21]. Among them, bevacizumab is currently widely used in advanced ovarian cancer and can significantly prolong the PFS of patients [22]. It is the first-line maintenance treatment drug approved by the National Comprehensive Cancer Network (NCCN) guidelines for advanced ovarian cancer and the first drug with Food and Drug Administration (FDA) certification that can be used for platinum resistance anti-angiogenesis therapy. Bevacizumab has contributed greatly to the treatment of recurrent ovarian cancer. Tyrosine kinase inhibitors (TKIs) are another common type of antitumour drug and are AIs that can specifically inhibit the intracellular tyrosine kinase activity of VEGFR. They can be used as tyrosine analogues or in the form of adenosine triphosphate to compete with tyrosine kinases to bind VEGFR kinase domains, thereby blocking the activity of tyrosine kinases, inhibiting angiogenesis and promoting cell apoptosis; moreover, TKIs have the advantages of high selectivity and high oral bioavailability [23, 24].

Apatinib mesylate (apatinib) is an orally administered tyrosine kinase VEGFR2 inhibitor. There is direct evidence that apatinib can normalize tumour blood vessels, correcting tumour blood concerns and the hypoxic tumour microenvironment. It can reduce the epithelial-mesenchymal transition of EOC cells by inhibiting the JAK/STAT3, PI3K/Akt and Notch signalling pathways and block tumour-induced angiogenesis [25]. The normalization of tumour blood vessels and the microenvironment is beneficial for improving the delivery of other related chemotherapeutics to tumours. There are many reports on the use of apatinib alone or in combination with other conventional chemotherapeutics for EOC, showing encouraging antitumour activity and tolerable toxicity. Miao et al. [26] described the efficacy of apatinib for platinum-resistant recurrent EOC. Twenty-nine patients with platinum-resistant EOC who relapsed after standard treatment were administered 500 mg apatinib daily. The median PFS was 5.1 months. The related AEs mainly included hand-foot syndrome, hypertension, gastrointestinal tract reactions, etc. In the AEROC study [27], apatinib combined with oral etoposide had a good effect on platinum-resistant or platinum-refractory ovarian cancer, with an ORR of 54.3% and a PFS of 8.1 months (95% CI 2.8–13.4). The most common grade 3–4 AEs included blood-related toxicity, fatigue, and mucositis. There were no deaths related to treatment, and all AEs were controllable and tolerable. Therefore, apatinib combined with etoposide in the treatment of platinum-resistant relapsed ovarian cancer shows a breakthrough effect and is expected to break its treatment bottleneck. The above studies have confirmed that apatinib can achieve good efficacy in advanced recurrent ovarian cancer, but the above studies were all single-arm studies. This article aimed to study the efficacy and safety of apatinib alone or combined with chemotherapy in advanced recurrent ovarian cancer and analyse its related factors affecting prognosis.

A total of 108 patients were enrolled in this study. The oral targeted drug apatinib was administered to the experimental group. The DCR of the apatinib group was significantly improved compared with that of the control group (66.67% vs 44.46%), *P* < 0.05. For the ORR, there was no significant difference between the two groups (30.56% vs 16.67%), *P* > 0.05. However, in the trial, compared with conventional chemotherapy, apatinib significantly prolonged PFS (6.0 months vs 3.6 months) and OS (15.8 months vs 9.2 months), and the differences were statistically significant (*P* < 0.05). Apatinib reduces the risk of disease progression in patients with advanced recurrent EOC by 42% and the risk of death by 53% compared with the control group. The effect is significant. The results of the subgroup analysis showed that patients who took more than 3 courses of oral apatinib had a longer median PFS (7.2 months vs 2.7 months) and median OS (28.3 months vs 10.0 months) than patients who took a shorter course of drug treatment, and the difference was significant. Taking apatinib for more than 3 courses significantly reduces the risk of disease progression, recurrence and death in patients. In addition, the results of the study suggest that the median PFS times of patients with tumour recurrence inside or outside the pelvis were 10.0 months and 4.0 months, respectively. Local tumour recurrence significantly reduced the risk of disease progression [HR 0.40 (95% CI 0.19–0.83), *P* = 0.016].

In the analysis of related single factors or multiple factors that affect PFS and the prognosis of patients, the results show that the course of treatment and the changes in tumour serum CA125 levels are independent risk factors that affect PFS. The independent risk factors affecting OS include the course of drug treatment after tumour recurrence, the location of the lesion at the time of recurrence, and whether apatinib was used. The tumour marker CA125 is a specific detection index for EOC, which basically shows abnormal conditions in advanced EOC. It is widely used in monitoring the prognosis of recurrent ovarian cancer [6, 28]. The Gynecological Cancer Society (GCIG) believes that the treatment effect of apatinib is good, which means that the CA125 level drops by at least 50% after treatment [17, 18]. The results of this article show that a good decrease in CA125 after treatment can reduce the risk of disease progression by 50% [HR 50 (95% CI 0.33–0.76), *P* = 0.001]. The treatment course of apatinib and the length of the treatment course of chemotherapeutics are independent risk factors that affect the patient’s PFS and prognosis; that is, after tumour recurrence, adherence to drug treatment is important to reduce the risk of recurrence and death. At the same time, the choice of treatment, improving the patient’s quality of life and improving the patient’s compliance with doctor’s advice are also the current problems facing clinical work. In this study, apatinib was another independent risk factor affecting the prognosis of patients (B: -0.968, HR: 0.380, 95% CI: 0.217–0.644, *P* = 0.001). Compared with conventional chemotherapy, it can significantly reduce the risk of death in patients with platinum-resistant recurrent ovarian cancer.

The results of the analysis of drug safety showed that the most common adverse reactions in the experimental group were hypertension, hand-foot syndrome and oral mucosal ulcers, which were similar to those caused by apatinib in other solid tumours [23, 29]. In this study, using grade 3–4 adverse reactions as a single-factor to analyse the impact on survival prognosis in the experimental group and the control group, the results showed that the difference was not statistically significant (*P* > 0.05). Subgroup analysis showed that when the dose of apatinib was reduced, there was no difference in the PFS and OS of the patients (*P* > 0.05). Therefore, there is no significant correlation between the AEs of apatinib and its efficacy. In addition, the experimental group and the control group had similar severe AEs. Among them, the grade 3–4 AEs in the experimental group were hypertension, and the total incidence of hand-foot syndrome was 22.22 and 16.67% in the apatinib and control groups, respectively, which was the main reason for reducing the amount of apatinib in this trial. One patient had no history of underlying hypertension, had a monitored systolic blood pressure of up to 220 mmHg during medication, adjusted the dosage of apatinib to 250 mg per day and combined use of dual antihypertensive drugs. Blood pressure control was acceptable, and no related lethal cardiovascular accidents occurred. Compared with the control group, there was no bone marrow suppression caused by related chemotherapy toxicity, the overall process of medication was safe and convenient, the AEs were controllable and tolerable, and the fear of seeking medical treatment was reduced, which is conducive to improving patients’ compliance with medical treatment and their health psychology.

In summary, the application of apatinib as a single agent or combined with chemotherapy in advanced recurrent EOC has shown gratifying efficacy and can be used as a clinical treatment decision direction. However, due to the small sample size in this study, the control group used different treatment options. The course of chemotherapy varied, and all data were retrospectively analysed. Thus, there may be sample selection and measurement biases in the analysis results due to differences in records during the follow-up of medical records. Therefore, this study is conducting a phase II multicenter open-label randomized controlled prospective clinical trial to further confirm its clinical value. The trial was evaluated according to 1 (apatinib + second-line chemotherapy): 2 was enrolled (second-line chemotherapy), and the inclusion criteria were age ≥ 18 years and ≤ 70 years old, apatinib combined with second-line chemotherapy (doxorubicin liposome or Cyclophosphamide or topotecan or nab-taxane) versus the single second-line chemotherapy control described above in patients with epithelial ovarian, fallopian tube, or primary peritoneal cancer that recurred within less than 6 months of last platinum treatment The safety and efficacy of apatinib is 250–500 mg/time, qd, orally, until disease progression or intolerable toxicity, and the primary endpoint is to assess objective response rate (ORR; complete and partial responses), and secondary endpoints were DOR, PFS, OS and safety. This study is currently enrolling.

## Data Availability

The data during the current study are available from the corresponding author on reasonable request.
